# Bta-miR-34b controls milk fat biosynthesis via the Akt/mTOR signaling pathway by targeting RAI14 in bovine mammary epithelial cells

**DOI:** 10.1186/s40104-021-00598-8

**Published:** 2021-07-19

**Authors:** Yujuan Wang, Xiaoyu Wang, Meng Wang, Li Zhang, Linsen Zan, Wucai Yang

**Affiliations:** College of Animal Science and Technology, Northwest A&F University, Xianyang, 712100 Shaanxi China

**Keywords:** Akt/mTOR signaling pathway, Bovine mammary epithelial cells, Milk fat, MiR-34b, RAI14

## Abstract

**Background:**

The biosynthesis of milk fat affects both the technological properties and organoleptic quality of milk and dairy products. MicroRNAs (miRNAs) are endogenous small non-coding RNAs that inhibit the expression of their mRNA targets and are involved in downstream signaling pathways that control several biological processes, including milk fat synthesis. miR-34b is a member of the miR-34 miRNA cluster, which is differentially expressed in the mammary gland tissue of dairy cows during lactation and dry periods. Previous studies have indicated miR-34b is a potential candidate gene that plays a decisive role in regulating milk fat synthesis; therefore, it is important to focus on miR-34b and investigate its regulatory effect on the biosynthesis of milk fat in bovine mammary epithelial cells (BMECs).

**Results:**

In this study, elevated miR-34b levels reduced milk fat synthesis, upregulated 1,999 genes, and downregulated 2,009 genes in BMECs. Moreover, Kyoto Encyclopedia of Genes and Genomes (KEGG) analysis of differentially expressed genes suggested that miR-34b may play an inhibitory role in milk fat synthesis via the protein kinase B (Akt)/mammalian target of rapamycin (mTOR) signaling pathway by reducing phosphorylation levels. Notably, the mTOR activator MHY1485 rescued the inhibitory effect of miR-34b. Furthermore, we demonstrated that retinoic acid-induced protein 14 (RAI14) is a target of miR-34b via TargetScan and immunofluorescence assays. RAI14 mRNA and protein levels were significantly decreased by the miR-34b mimic and increased by the miR-34b inhibitor. Moreover, the reduction in RAI14 levels led to the inhibition of the Akt/mTOR signaling pathway.

**Conclusions:**

Overall, our results identified a miR-34b-RAI14-Akt/mTOR regulatory network, while also providing a theoretical basis for the molecular breeding of dairy cows.

## Introduction

Milk fat biosynthesis has received considerable attention because of its influence on the technological properties and organoleptic quality of milk and dairy products [1]. Bovine mammary epithelial cells (BMECs) are highly active in the biosynthesis of triacylglycerols (TAGs), which account for nearly 95% of the fat content in milk, and are therefore considered an indicator of milk fat content [2]. The synthesis and secretion of milk fat are regulated by various factors, such as diet, hormones, and via gene networks [3–5]. In this study, we mainly focused on exploring the gene network that regulates synthesis of milk fat, and clarified a miRNA–mRNA signaling pathway network which plays an important role in milk fat synthesis.

miRNAs are a class of endogenous non-coding single-stranded RNA molecules with a length of 18–25 oligonucleotides, and have received considerable attention as they play an important role in cells by completely or incompletely binding with the 3′-UTR of target mRNAs in two phases: post-transcriptionally, and before translation [6]. miRNAs thereby have an important role in various cellular processes such as cell proliferation, differentiation, programmed apoptosis and cell death [7, 8]. Additionally, these small RNAs may be a crucial factor in mammogenesis through their regulation of downstream genes. Tanaka et al. [9] found significant differences in the expression of miRNAs during different stages of mouse mammary epithelial cell differentiation. Solexa sequencing and bioinformatic analysis of dairy goat mammary gland tissues revealed 697 conserved miRNAs were significantly differentially expressed between dry and lactation periods [10].

Moreover, miRNAs appear to affect milk fat metabolism and biosynthesis [11]. For instance, chi-miR-183 plays an important regulatory role in milk fat metabolism in goat mammary epithelial cells by targeting *MST1* [12]. In BMECs, bta-miR-181a plays a decisive role in regulating milk fat biosynthesis by inhibiting *ACSL1* [13], while miRNA-106b regulates milk fat metabolism via *ABCA1* [14]. Although many miRNAs involved in the regulation of milk fat synthesis have been identified, their mechanisms of action require further investigation. Additionally, the identification of novel important miRNAs involved in milk fat synthesis, and the exploration of their molecular mechanisms, are also required.

miR-34b is a member of the miR-34 miRNA cluster, which is differentially expressed in the mammary gland tissue of dairy cows during lactation and dry periods [15, 16], indicating miR-34b is a potential candidate gene that could play a decisive role in the regulation of milk fat synthesis. Therefore, in this study, we focused on miR-34b and investigated its regulatory activity on milk fat biosynthesis in BMECs.

## Materials and methods

### Cell preparation

BMECs were isolated from breast tissue derived from Holstein cows during mid-lactation, according to previously published protocols [17, 18]. Purified cells were cultured in complete growth medium containing 90% Dulbecco’s modified Eagle’s medium (DMEM)/F-12 (Sigma-Aldrich), 10% fetal bovine serum (FBS; Sigma-Aldrich), and 100 μg/mL penicillin-streptomycin (Gibco) at 37 °C in a humidified incubator with 5% CO_2_. The cells were passaged in 6-well cell culture plates using 0.25% trypsin and grown to 80% confluence in 10-cm cell culture dishes. The medium was then discarded, and cells were cultured for 48 h in a lactogenic medium (complete medium supplemented with 5 μg/mL insulin, 2 μg/mL prolactin, and 1 μg/mL hydrocortisone).

To investigate the role of miR-34b in milk fat synthesis in BMECs, a set of cells were treated with 50 nmol/L miR-34b mimic and 50 nmol/L miR mimic NC was used as its negative control. Correspondingly, the other set of cells were transfected with 200 nmol/L miR-34b inhibitor and 200 nmol/L miR inhibitor NC was used as its negative control. All the miR-34b mimic/inhibitor and their respectively negative control were designed and compounded by RiboBio (RiboBio Co., LTD). Moreover, 15 μmol/L of the mTOR signaling pathway-specific agonist MHY1485 (Sigma-Aldrich) was used to reverse the inhibitory effects of the miR-34b mimic (RiboBio) on milk fat synthesis to clarify the role of the mTOR signaling pathway in this process. To determine the underlying mechanism of the target mRNA in milk fat synthesis, cells were transfected with 100 nmol/L of a small interfering RNA (siRNA) against retinoic acid-induced protein 14 (si-RAI14; Songon Biotech Co., Ltd.). Three biological replicates for each treatment condition were used, and the cells of each treatment groups were collected at 48 h post-treatment for further experiments.

### RNA-seq and real-time PCR

Total RNA was extracted using the TRIzol reagent (Invitrogen) and reverse-transcribed with a Reverse Transcription Kit (TaKaRa) or the PrimeScript RT Reagent Kit (Takara, Dalian, China) for subsequent analysis of miRNA or mRNA expression, respectively. All RNA sequencing processes and methods were conducted by Novogene Sequencing Company (Beijing, China). qRT-PCR was performed using the miRcute miRNA qPCR Detection Kit (Tiangen) or SYBR Premix ExTaq II (TaKaRa) on a 7500 Real-Time PCR System (Applied Biosystems Inc., Foster City, CA, USA) to quantify miR-34b and mRNA levels, respectively. U6 and UXT were used as internal control genes for the quantitative analysis of miRNA and mRNA levels, respectively [19]. Primers (Tables 1 and 2) were purchased from TSINGKE Biological Technology (Xi’an, P.R. China). The relative expression of miR-34b and mRNA was calculated using the 2^−ΔΔCT^ method.

**Table 1:** Primers for mRNA quantitative real-time PCR

Genes	Primer sequence (5'→3')	Annealing temperature
*UXT*	F: TAGCCACCCTCAAGTATGTTCG R: CGAGGTAGGAGGACAGGAGT	61 °C
*PPARγ*	F: AAAGGAGAGCCTGAACTTGGAG R: TCTGAACTGTGCTGTGGCAA	61 °C
*FASN*	F: CCCTGAATGTGAGGCAGTGTG R: TTAGCTGTGGTGAGGAGCCA	61 °C
*FABP4*	F: GGACAGCAAGGAGACCTACAA R: GTTTGAGATGTTCGGGGAGGA	61 °C
*CEBPα*	F: CGAAGTGTAGGAACCGGCGA R: CACAAACTCCAGACGTTCCTTC	61 °C
*CEBPβ*	F: TGGTGAATAGTGCTGCCCAT R: GGTGGTAGTTGTGGAAGCCC	61 °C
*Akt*	F: TCACCATTACGCCACCTGAC R: TCCTCTCCATCCTGTGTTGG	61 °C
*RAI14*	F: AAGCTCCACCACCTCCTATCA R: GTATGGAACTAATCTCAGCCTTGAA	61 °C
*mTOR*	F: TGGACACCAACAAGGACGAC R: TCCCACTGACCTAAACCCCA	61 °C
*4E-BP1*	F: GTTCCTGATGGAGTGTCGGA R: AACTGTGACTCTTCACCGCCT	61 °C
*P70S6K*	F: ATCACCAAGGTCACGTCAAAC R: TGCTCCCAAACTCCACCAAT	61 °C
*S100G*	F: CTCCAGAAGAACTGAAGGGCA R: CAAAAAGCTCATCGAGGGTGC	61 °C
*PABPC1*	F: GGTGCCAGACCTCATCCATT R: TCGACATGACTCGTGGAACC	61 °C
*TFPI2*	F: ATGGTAACACAGGCAAGCGA R: AAATCAGGGAGAGCAACCCC	61 °C
*PRICKLE1*	F: GCGCCGGAAGGAATCGAAC R: CAGCCCGAGTCATCATCCG	61 °C
*SYNE2*	F: GTGGTTTGCAGCTTTCCGAG R: CTGTTGGTCTTCAGCGGGAA	61 °C
*IGF2*	F: GACCTTGAGGACGAGGAGGT R: CTGAGCAGGTGGGGATCAAG	61 °C
*IVNS1ABP*	F: AGGCAAAGAGGCACATGGGA R: GCCACCAGGCAAACTGATTTC	61 °C
*SGK1*	F: TCTGGCGATGACGGTGAAAAC R: TCATTCAGGCCCATCCTTCTC	61 °C
*PLSCR2*	F: ACCAAAGAAGGACACACTTAGAGA R: CACTTGAGGGATCTGAAAACTACT	61 °C
*TNS1*	F: ACCAGCTTCTTGCCATCCAC R: CGGAGCAGTCTCAGTGTTGA	61 °C
*MDH1*	F: CTGCAAAGGCCATCTGTGAC R: ACGGGGAACGAGTAAAGCAG	61 °C
*S100A10*	F: TAATCGCTGGGCTCACCATC R: AAGCTGTGGGACAGAGTCCT	61 °C
*TM4SF1*	F: CGATCCTCTTGGCACTTGGT R: TGGCGAGAGGAACAATGACC	61 °C
*PSPH*	F: CCCCTTGCAGTCGGAACTT R: TGGATGACGGTGCTATCGAC	61 °C
*BTBD11*	F: GGCCCGAACCTGTAAACTGT R: TCTGCAACAGCATCTCAGGG	61 °C
*SYT11*	F: CCACCAGATACCTTCCCCCT R: GGTGATCTCAGCCATGTCGT	61 °C
*TCIM*	F: CCGAAGAATCCCCCATCCAT R: GCCCACAGCTTTCTTACGTG	61 °C
*TAGLN2*	F: ACTCTCCGACCCACGTGAA R: TGCCATCCTTGAGCCAGTT	61 °C
*RCN1*	F: TGTCAGAACGGGAGCAGTTT R: GTGGTCGTAGTCTTGGGGAAG	61 °C
*NDFIP2*	F: AGAAACAGAGGCGGAAGAGTC R: CTGGTGGTGATCCATCCTGC	61 °C
*ANXA5*	F: GCCATGGCACAGGTTCTCAG R: TGCGGGATGTCAACAGAGTC	61 °C
*TRPM6*	F: TGCAGGTGCCAGTCATAACA R: CAGGATGTCGTTCAGCTCCTT	61 °C
*RCAN1*	F: TAGCTCCCTGATTGCCTGTGT R: ATGTAGCTGGAGTCTGGCATC	61 °C
*COL5A2*	F: TGGGGCACTATGATGACAGC R: ATCATCACAAGTCCGGGCAG	61 °C

*F* forward, *R* reverse

**Table 2:** Primers for miRNA quantitative real-time PCR

Genes	Primer sequence (5'→3')	Annealing temperature
*U6*	F: GCTTCGGCAGCACATATACT R: TTCACGAATTTGCGTGTCAT	61 °C
*miR-34b*	GGGGAGGCAGTGTAATTAGCTGATTG	61 °C

*F* forward, *R* reverse

### Protein extraction and western blotting

BMECs were collected using 0.25% trypsin and lysed in RIPA buffer (Solarbio, China) supplemented with 1% phenylmethanesulfonyl fluoride (PMSF; Pierce, USA) and 1% phosphatase inhibitor cocktail (Roche). Western blotting was performed as previously described [4], using primary antibodies against β-actin (mAbcam 8226, 1:1,000, Abcam), peroxisome proliferator-activated receptor gamma (PPARγ; EP4394(N), 1:1,000, Abcam), fatty acid synthase (FASN; ab99359, 1:2,000, Abcam), protein kinase B (Akt, #9272, 1:500, CST), pho-Akt (Ser473, 1:500, CST), mTOR (5536 T, 1:1,500, Univ), pho-mTOR (5536 T, 1:1500, Univ), ribosomal protein S6 kinase B1 (P70S6K; 2708 T, 1:1,500, Univ), pho-P70S6K (9234 T, 1:1,500, Univ), Eukaryotic Translation Initiation Factor 4E-Binding Protein 1 (4E-BP1; 9644 T, 1:1500, University), Pho-4E-BP1 (2855 T, 1:1,500, Univ), and RAI14 (EPR8518, 1:1,000, Abcam).

### Oil red O staining

At 48 h post-treatment, BMECs were washed three times with phosphate-buffered saline (PBS) and then fixed using 4% paraformaldehyde. Lipid droplets were stained with Oil Red O. The cells were then washed and placed under a microscope to evaluate the number of lipid droplets according to previously published methods [20].

### Cellular triacylglycerol assay

BMECs were washed three times with PBS at 48 h post-treatment and then collected using 0.25% trypsin. The cells were then lysed with cell lysis buffer (Applygen Technologies Inc.), and the supernatant was collected and heated at 70 °C for 10 min. The mixture was then centrifuged at 5,000 r/min for 5 min, and the supernatant was used to determine the TAG content according to the manufacturer’s recommended protocol (Applygen Technologies, Beijing, P.R. China).

### Luciferase assay

The target mRNA of miR-34b was predicted by TargetScan (http://www.targetscan.org) and preliminarily determined by aligning the miR-34b mature sequence and the 3′-UTR sequence of RAI14. Wild-type (WT) and mutation-type (MUT) RAI14 were cloned into the pmirGLO dual-luciferase miRNA target expression vector (Promega, Madison, WI, USA) using the *Xho*I and *Not*I restriction sites. BMECs were cultured in 12-well dishes and transfected when they reached 70% confluence. After 48 h, fluorescence intensity was measured using the Dual-Glo Luciferase Assay System Kit (Promega) according to the manufacturer’s instructions. All experiments were performed in triplicate. Firefly luciferase activity was normalized to internal Renilla luciferase activity.

### Statistical analyses

All statistical analyses and visualization were performed using GraphPad Prism 7.00 software. Significant differences between the two groups were determined using a two-tailed Student’s t-test. All results are presented as mean ± standard error of the mean (SEM), and a *P*-value of < 0.05 was considered statistically significant. Additionally, the padj was used to represent the *P*-value, which was adjusted via multiple hypothesis testing corrections.

## Results

### miR-34b controls milk fat synthesis in BMECs

**Fig. 1:**
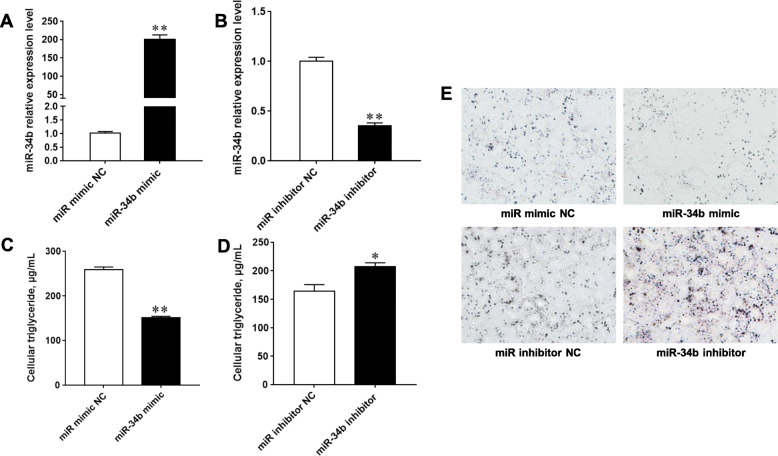
Effects of the bta-miR-34b mimic/inhibitor on milk fat synthesis in BMECs. **a** and **b** miR-34b relative expression levels. **c** and **d** Effects of the miR-34b mimic/inhibitor on TAG accumulation. **e** Representative images of Oil Red O stained BMECs. Values are presented as the mean ± SEM. *, *P* < 0.05; **, *P* < 0.01. BMECs = bovine mammary epithelial cells, TAG = intracellular triglyceride, NC = negative control

A miR-34b mimic and inhibitor were used to enhance or inhibit the regulatory role of miR-34b in BMECs. With regards to their overexpression and interference efficiency, the expression level of miR-34b in BMECs was increased 200-fold after treatment with the miR-34b mimic, and decreased by 65% after treatment with the miR-34b inhibitor, compared to corresponding negative controls (NCs) (Fig. 1a and b).

**Fig. 2:**
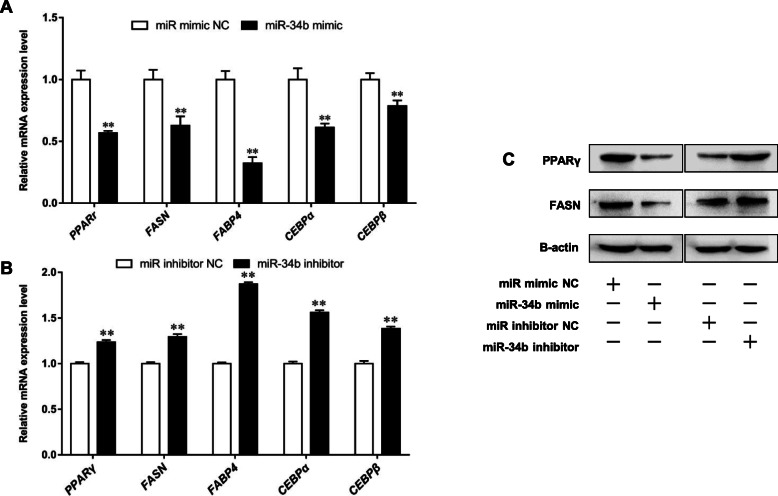
Effects of the bta-miR-34b mimic/inhibitor on lipid synthesis-related genes in BMECs. **a** and **b** mRNA expression levels of *PPARγ*, *FASN*, *CEBPα*, *CEBPβ*. **c** Protein expression levels of *PPARγ* and *FASN*. Values are presented as the mean ± SEM. *, *P* < 0.05; **, *P* < 0.01. BMECs = bovine mammary epithelial cells, TAG = intracellular triglyceride, NC = negative control

The cellular TAG assay and Oil Red O staining were used to assess the effect of miR-34b on milk fat synthesis in BMECs. Our results showed that the miR-34b mimic decreased TAG content and droplet numbers in BMECs, while treatment with the miR-34b inhibitor markedly increased TAG content and droplet numbers in BMECs when compared to their corresponding NCs (Fig. 1c-e). Additionally, we investigated the effect of miR-34b on lipid synthesis-related genes by determining the mRNA expression levels of *PPARγ*, *FASN*, fatty acid-binding protein 4 (*FABP4*), CCAAT enhancer-binding protein alpha (*CEBPα*), and *CEBPβ,* as well as the protein expression levels of PPARγ and FASN. The results of the qPCR and western blot analyses showed that the miR-34b mimic significantly decreased the mRNA expression levels of *PPARγ* (*P* < 0.01), *FASN* (*P* < 0.01), *FABP4* (*P* < 0.01), *CEBPα* (*P* < 0.01), and *CEBPβ* (*P* < 0.01), as well as suppressed PPARγ and FASN protein expression levels in BMECs (Fig. 2a, c). In contrast, treatment with the miR-34b inhibitor significantly increased the mRNA expression levels of *PPARγ* (*P* < 0.01), *FASN* (*P* < 0.01), *FABP4* (*P* < 0.01), *CEBPα* (*P* < 0.01), and *CEBPβ* (*P* < 0.01)*,* and elevated PPARγ and FASN protein expression levels in BMECs (Fig. 2b, c). Overall, these results indicate that miR-34b plays an important role in the inhibition of milk fat synthesis in BMECs.

### miR-34b suppresses milk fat synthesis by regulating a key milk fat synthesis-related pathway

**Fig. 3:**
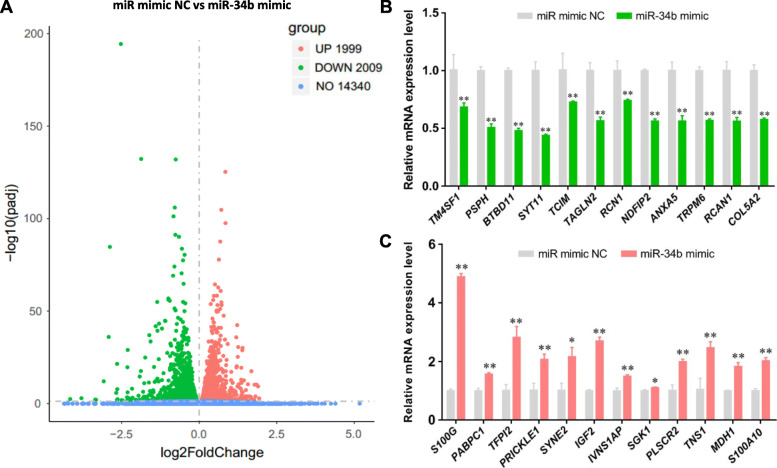
Differentially expressed genes between BMECs treated with 50-nM miR-34b mimic and NCs. **a** Volcano plot of the transcriptome of BMECs (*n* = 3). **b** qPCR analysis of the genes with the most significant differences in the Volcano plot. Values are presented as the mean ± SEM. *, *P* < 0.05; **, *P* < 0.01. BMECs = bovine mammary epithelial cells, NC = negative control

**Fig. 4:**
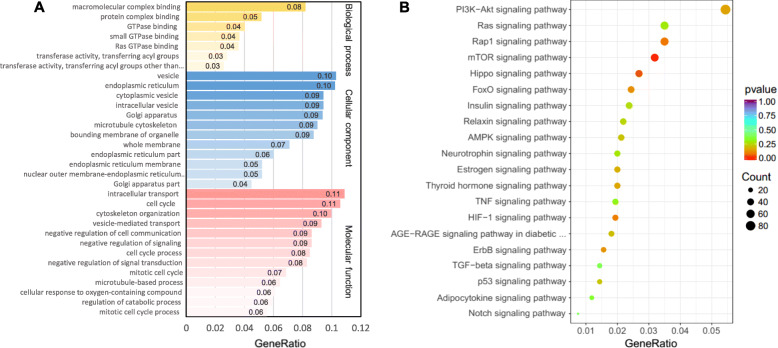
GO and KEGG analysis of the differentially expressed genes in BMECs. **a** GO analysis of the differentially expressed genes (*n* = 3). **b** The most significant enrichment signaling pathway of the KEGG analysis (*n* = 3). BMECs = bovine mammary epithelial cells, NC = negative control, GO = Gene Ontology

To determine the signaling pathways involved in miR-34b-induced inhibition of milk fat synthesis in BMECs, the transcriptomes of BMECs transfected with a miR-34b mimic and miRNA mimic NCs were analyzed. The results showed that 4008 genes were significantly altered (−log10 padj *>* 1.30) and that the expression of 49.88% (1999/4008) of the genes was increased in miR-34b mimic-treated cells, whereas 50.12% (2009/4008) were decreased (Fig. 3a). Moreover, quantitative analysis of gene expression levels via qPCR of the genes with the most significant differences (according to padj value) in expression confirmed the accuracy of the sequencing results (Fig. 3b–c). Gene Ontology (GO) analysis showed that 153 of these genes are related to the biological processes occurring in the endoplasmic reticulum, which is the main site of milk fat synthesis (Fig. 4a). Furthermore, Kyoto Encyclopedia of Genes and Genomes (KEGG) analysis of the DEGs indicated that the phosphatidylinositol-3 kinase (PI3K)-Akt signaling pathway, which had the most significant GeneRatio (GeneRatio = 0.054), and the mTOR signaling pathway, which had the most notable *P*-value (*P* = 0.0085), were involved in the miR-34b-induced regulation of milk fat synthesis (Fig. 4b).

### miR-34b suppresses milk fat biosynthesis via the Akt/mTOR pathway

**Fig. 5:**
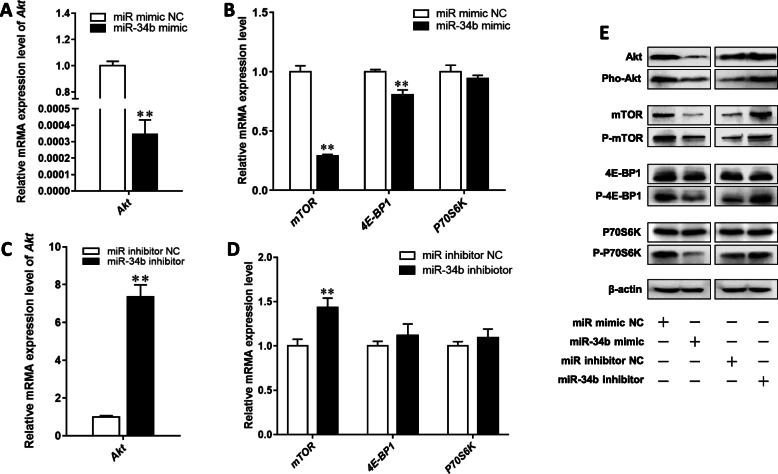
Effects of the miR-34b mimic and inhibitor on the Akt/mTOR signaling pathway in BMECs. **a**-**d** The relative mRNA expression level of *Akt*, *mTOR*, *4E-BP1*, and *P70S6K.* **e** The total protein and phosphorylated protein expression level of Akt, mTOR, 4E-BP1, and P70S6K. Values are presented as the mean ± SEM. *, *P* < 0.05; **, *P* < 0.01. BMECs = bovine mammary epithelial cells, NC = negative control

**Fig. 6:**
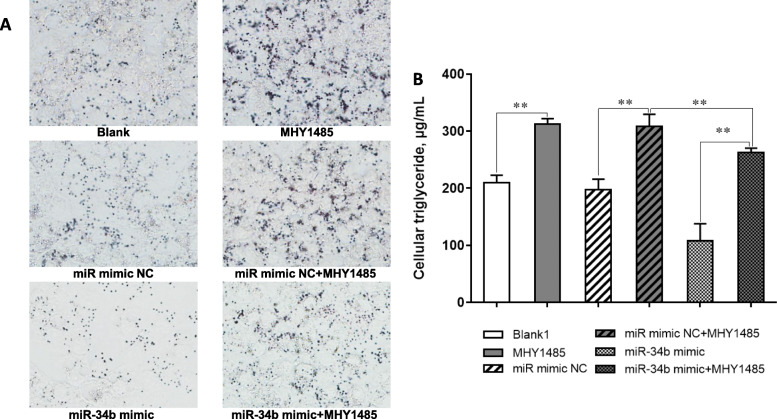
Treatment with MHY1485 reverses the inhibitory effect of miR-34b mimic on milk fat synthesis. **a** Representative images of Oil Red O stained BMECs. **b** Effects of the miR-34b mimic and MHY1485 on TAG accumulation. Values are presented as the mean ± SEM. *, *P* < 0.05; **, *P* < 0.01. BMECs = bovine mammary epithelial cells, TAG = intracellular triglyceride, NC = negative control

Activation of the Akt/mTOR signaling pathway plays a crucial role in the regulation of milk fat synthesis [4, 21]. Therefore, we investigated whether miR-34b exerts its biological functions by inhibiting the Akt/mTOR pathway. miR-34b overexpression in BMECs led to a remarkable decrease in Akt and mTOR mRNA, protein, and phosphorylated protein expression levels, resulting in reduced 4E-BP1 mRNA and protein expression levels, as well as 4E-BP1 and P70S6K phosphorylated protein levels (Fig. 5a, b, e). Conversely, miR-34b knockdown in BMECs robustly enhanced the mRNA, protein, and phosphorylated protein expression levels of Akt and mTOR, resulting in increased levels of phosphorylated 4E-BP1 and P70S6K (Fig. 5c–e). These results suggest that miR-34b overexpression suppresses the Akt/mTOR pathway in BMECs. Next, we investigated the effects of a specific activator of the mTOR pathway on miR-34b-induced inhibition of milk fat synthesis in BMECs. The effectiveness of the mTOR activator, MHY1485, was first confirmed (Fig. 6a). Further investigation via Oil Red O staining and the TAG assay showed that the inhibitory effect of miR-34b on milk fat synthesis could be reversed by MHY1485 (Fig. 6b).

### miR-34b directly targets RAI14 in BMECs

**Fig. 7:**
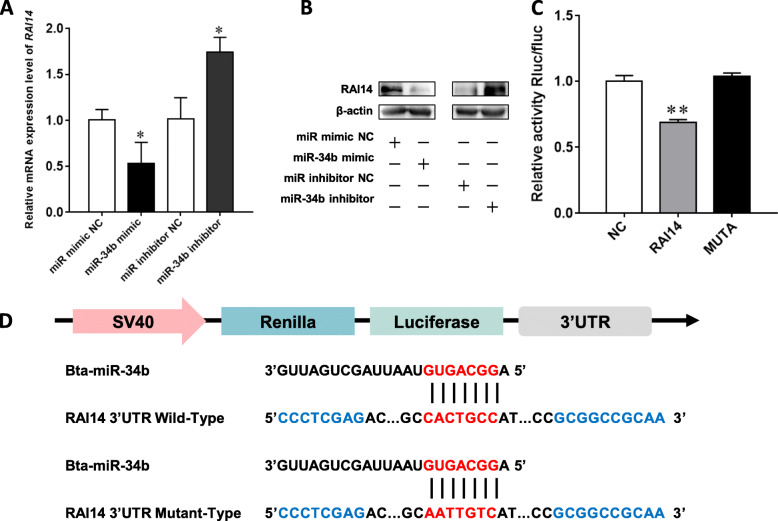
RAI14 is the target gene of miR-34b in BMECs. **a**, **b** The relative mRNA and protein expression levels of RAI14 in BMECs following transfection with a miR-34b mimic or inhibitor. **c** The BMECs were co-transfected with the miR-34b mimic and a luciferase reporter containing a fragment of the RAI14 3′-UTR harboring either the miR-34b binding site (RAI14–3′-UTR WT) or a mutant (RAI14–3′-UTR MUTA). **d** Target site of miR-34b in the RAI14 3′-UTR and the construction of the luciferase (Luc) expression vector fused with the RAI14 3′-UTR. Values are presented as the mean ± SEM. *, *P* < 0.05. **, *P* < 0.01. Rluc = Renilla luciferase, fluc = firefly luciferase, WT = wild-type, MUTA = mutation type, NC = negative control

To clarify the mechanism underlying the effects of miR-34b on the Akt/mTOR pathway, we first used TargetScan to predict the target mRNA of miR-34b and found that RAI14 has a miR-34b binding site in its 3′-UTR (Fig. 7d). RAI14 mRNA and protein levels were measured to determine the regulatory effects of miR-34b on RAI14. The results showed that the miR-34b mimic significantly decreased RAI14 mRNA and protein levels (*P* < 0.01), while the miR-34b inhibitor had the opposite effect (Fig. 7a, b). Furthermore, the luciferase reporter system was used to confirm whether RAI14 is a direct target gene of miR-34b. The results showed that the miR-34b mimic inhibited the standardized luciferase activity by 31.31% (*P* < 0.01), whereas the activity returned to normal levels in the MUT group (Fig. 7c). These results indicate that miR-34b directly binds to the 3′-UTR of RAI14, thereby inhibiting its activity.

### RAI14, as a target gene of miR-34b, participates in the regulation of the downstream Akt/mTOR signaling pathway

**Fig. 8:**
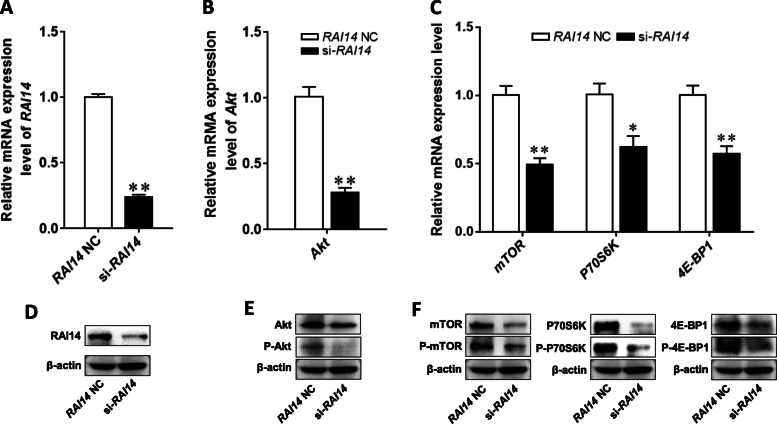
Effects of RAI14 on the Akt/mTOR signaling pathway in BMECs. **a** The transfection efficiency of si-RAI14. **b**-**f** Effects of si-RAI14 on the mRNA, protein, and phosphorylated protein levels of Akt, mTOR, 4E-BP1, and P70S6K. Values are presented as the mean ± SEM. *, *P* < .05; **, *P* < 0.01. BMECs = bovine mammary epithelial cells, NC = negative control

To clarify the regulatory relationship between the target gene of miR-34b and the Akt/mTOR pathway, BMECs were transfected with a RAI14 siRNA, after which the activity of the Akt/mTOR signaling pathway was investigated. The optimal transfection concentration of siRNA in BMECs was 100 nmol/L, and the mRNA and protein levels of RAI14 were shown to decrease in the RAI14 siRNA group compared to the NC group (Fig. 8a, d). The mRNA, protein, and phosphorylated protein levels of Akt, mTOR, 4E-BP1, and P70S6K were significantly decreased after treatment with RAI14 siRNA (Fig. 8b, c, e, f). These results suggest that RAI14 silencing inhibits the activity of the Akt/mTOR pathway.

**Fig. 9:**
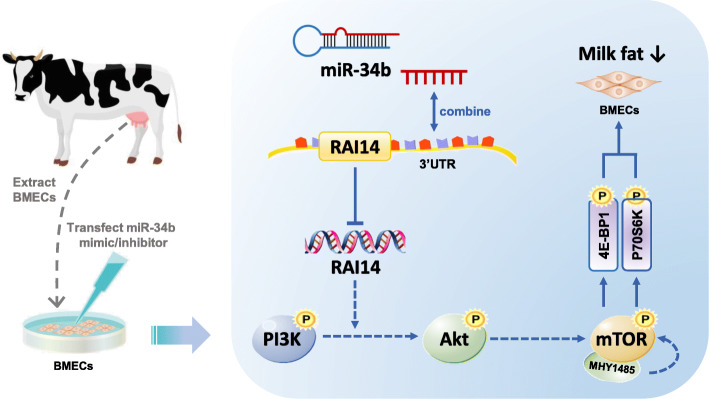
Path summary diagram of the mechanisms of action of miR-34b in BMECs. miR-34b first fuses with the 3′-UTR of RAI14 and then inhibits the Akt/mTOR signaling pathway, resulting in decreased synthesis of milk fat in bovine mammary epithelial cells

Taken together, these results indicate that RAI14 is an important connection between miR-34b and the Akt/mTOR pathway and that suppression of milk fat synthesis in BMECs by miR-34b is associated with RAI14 targeting, which subsequently inhibits the Akt/mTOR pathway (Fig. 9).

## Discussion

miRNAs have crucial regulatory effects on lipid biosynthesis in mammary glands, and may become potential regulatory targets during high- or low-fat milk production [22–25]. miR-34b is a potential regulatory target that may participate in lipid metabolism in bovine mammary cells [15, 16]. Nevertheless, little is known about the specific regulatory effects and molecular mechanisms of miR-34b in the regulation of milk fat synthesis. In this study, we revealed the role and underlying mechanism of miR-34b in reducing milk fat accumulation, which suggests that the regulation of miR-34b expression levels could be used to produce milk with high or low lipid content in the future.

In the initial experiment, upregulated miR-34b levels reduced milk fat synthesis, and KEGG analysis of DEGs revealed that miR-34b-induced inhibition of milk fat synthesis may occur via the PI3K/Akt/mTOR pathway. PI3Ks are lipid kinases that generate secondary messengers PIP3 [26]. The accumulation of PIP3 recruits Akt to the cell membrane where it is phosphorylated and activated by phosphoinositide-dependent kinase 1 and 2 [27]. Akt activation regulates numerous downstream effectors, such as mTORC1 [28]. mTOR is the core component of two different protein complexes, mTORC1 and mTORC2. mTORC1 and mTORC2 play important roles in regulating lipid metabolism and other cellular processes [29–31]. mTORC1 forms a stoichiometric complex with raptor, which promotes nutrient-stimulated signaling to the downstream effectors P70S6K and 4E-BP1, which controls the activation of translation and ribosome biosynthesis [32]. Considerable attention has been paid to the Akt and mTOR signaling pathways in mammary cells because they are involved in the regulation of lipid metabolism. For instance, Che et al. found that valine increases milk fat synthesis in the mammary glands of gilts by stimulating the AKT/mTOR pathway [21], while Schwertfeger et al. discovered that the expression of constitutively activated Akt in the mammary glands leads to excess lipid synthesis during pregnancy and lactation [33]. In addition, Wang et al. found that melatonin suppressed milk fat synthesis by reducing the activity of the mTOR signaling pathway in BMECs [4]. In this study, our results indicated that miR-34b suppressed the activity of the Akt/mTOR pathway by decreasing Akt and mTOR mRNA, protein, and phosphorylated protein expression levels, resulting in reduced 4E-BP1 mRNA and protein expression levels, as well as 4E-BP1 and P70S6K phosphorylated protein levels. Additionally, the mTOR pathway-specific agonist MHY1485 was used to rescue the effects of miR-34b-induced regulation of the Akt-mTOR axis on milk fat synthesis, and the results showed that the negative effects of miR-34b could be reversed by treatment with MHY1485. Therefore, these results show that the Akt/mTOR signaling pathway plays a very important role in the downstream regulatory pathway of miR-34b, which inhibits milk fat synthesis in BMECs.

Furthermore, to clarify the mechanism by which miR-34b and the Akt/mTOR pathway are connected, we used TargetScan to predict the target gene of miR-34b and determined a direct targeting relationship between miR-34b and its target gene RAI14 through a luciferase reporter system. Previous studies have shown that downregulation of RAI14 inhibits the activation of the Akt pathway in human stomach cells [34]. Therefore, we hypothesized that miR-34b may indirectly regulate the Akt/mTOR pathway by directly binding to the 3′-UTR of RAI14. Thus, we investigated Akt and mTOR mRNA, protein, and phosphorylated protein expression levels in mammary epithelial cells transfected with RAI14 siRNA. The results showed that Akt and mTOR mRNA, protein, and phosphorylated protein expression levels were downregulated by RAI14 siRNA. This suggests that downregulation of RAI14 is associated with the inhibition of the Akt/mTOR signaling pathway, and that RAI14 is an important mediator of miR-34b regulation which indirectly regulates the Akt/mTOR signaling pathway.

## Conclusions

In summary, we revealed that miR-34b decreased milk fat synthesis by targeting the RAI14-Akt/mTOR axis in BMECs. Thus, our results indicate that miR-34b may serve as a potential biomarker or therapeutic target for regulating synthesis of milk fat in the future (Fig. 9). These results may also provide an important reference for improving the production of beneficial milk components in dairy cows.

## Data Availability

The datasets used and/or analyzed during the current study are available from the corresponding authors upon reasonable request.

## Abbreviations

MST1: Macrophage stimulating 1
ACSL1: Acyl-CoA synthetase long chain family member 1
ABCA1: ATP binding cassette subfamily A member 1
PIP3: PI-3,4,5-trisphosphate
qRT-PCR: Real-time reverse transcription-polymerase chain reaction
